# Evaluation of E148Q and Concomitant AA Amyloidosis in Patients with Familial Mediterranean Fever

**DOI:** 10.3390/jcm10163511

**Published:** 2021-08-10

**Authors:** Zehra Serap Arici, Micol Romano, David Piskin, Ferhat Guzel, Sezgin Sahin, Roberta A. Berard, Mahmut I. Yilmaz, Erkan Demirkaya

**Affiliations:** 1 Department of Paediatric Rheumatology, Sanliurfa Training and Research Hospital, Sanliurfa 63250, Turkey; zehraserap@gmail.com; 2 Clinical Epidemiology, McMaster University, Hamilton, ON L8S 4L8, Canada; 3 Schulich School of Medicine & Dentistry, Department of Paediatrics, Division of Paediatric Rheumatology, University of Western Ontario, London, ON N6A 5W9, Canada; micol.dr.romano@gmail.com (M.R.); David.piskin@lhsc.on.ca (D.P.); Roberta.berard@lhsc.on.ca (R.A.B.); 4 Lawson Health Research Institute, London Health Sciences Center, London, ON N6C 2R5, Canada; 5 Schulich School of Medicine & Dentistry, Department of Epidemiology and Biostatistics, University of Western Ontario, London, ON N6A 3K7, Canada; 6 Molecular Genetics Laboratories, Genetics Research and Genome Center, Department of Research and Development, Gentera Biotechnology, Istanbul 34406, Turkey; frhtgzl@gmail.com; 7 Department of Pediatric Rheumatology, Basaksehir Cam ve Sakura City Hospital, University of Health Sciences, Istanbul 34480, Turkey; dr.sezginsahin@gmail.com; 8 Division of Nephrology, Center for Epigenetic Health Solutions, Ankara 06810, Turkey; mahmutiyilmaz@yahoo.com

**Keywords:** familial Mediterranean fever, AA amyloidosis, mortality, genotype, phenotype

## Abstract

The aim of the study was to compare the clinical phenotype of patients with familial Mediterranean fever (FMF)-related AA amyloidosis, according to the age of FMF diagnosis and E148Q genotype. Patients with biopsy-confirmed FMF-related AA amyloidosis were included in the study. Tel-Hashomer criteria were applied in the diagnosis of FMF. All patients had detailed baseline assessment of clinical features, renal functions, genetic testing, histopathological diagnosis of amyloidosis, and treatment received. Multiple comparisons were performed according to the age of diagnosis, disease phenotype, mutation, and mortality. Our study included 169 patients with a diagnosis of AA amyloidosis. There were 101 patients diagnosed with FMF < 18 years of age and 68 patients diagnosed who were ≥18 years of age. The three most common clinical manifestations were fever (84.6%), abdominal pain (71.6%), and arthritis (66.9%). The most common allele among FMF patients was M694V (60.6%), followed by E148Q (21.4%), and M680I (10.3%). The most frequent genotypes were M694V/M694V (45.0%), M694V/E148Q (14.8%), and E148Q/E148Q (11.2%) among 169 patients in our cohort. During the follow-up period, 15 patients (10 male, 5 female) died, of whom 14 had M694V homozygous genotype and one was homozygous for E148Q. Clinicians should be aware of patients with homozygous E148Q genotype for close monitoring and further evaluation. The possible relationship between E148Q and AA amyloidosis needs to be confirmed in other ethnicities.

## 1. Introduction

Amyloid A (AA) amyloidosis, previously known as secondary or reactive amyloidosis, is a long-recognized severe complication of some chronic inflammatory diseases. Familial Mediterranean fever (FMF) is an autosomal recessive autoinflammatory disease that occurs worldwide but predominantly affects populations of Mediterranean and Middle Eastern descent. The disease is characterized by recurrent episodes of fever and serositis, accompanied by elevated biomarkers of inflammation usually with childhood-onset. Acute attacks are followed by attack-free intervals, but subclinical inflammation may continue in some patients between the episodes [1].

In AA amyloidosis, the kidney is the most significantly affected organ, especially in untreated and non-compliant patients. The extent of renal damage defines the patients’ prognosis, and renal biopsy is the most common diagnostic test revealing amyloid deposits. The majority of reports on FMF-related AA amyloidosis are retrospective in nature or case series [2,3,4,5,6,7]. The pathogenesis and risk factors for amyloidosis in FMF remain partially understood. The development of AA amyloidosis is dependent on ethnicity and country of residence. In the pre-colchicine era, renal AA-amyloidosis was largely reported in patients of Turkish (67%) and Sephardic Jewish ancestry (26.5%) [8]. This may have been due to genetic and/or environmental factors that may impact disease severity and/or colchicine bioavailability. Many published reports from different countries showed that mutations in exon 10 such as M694V, M680I, and V726A are inevitably accompanied by typical clinical manifestations of FMF. However, the effect of mutations in exon 2, specifically the E148Q variant, on phenotype remains an unresolved conundrum [9].

In this study, we compared the clinical phenotype of patients with FMF-related AA amyloidosis, according to the age of FMF diagnosis and E148Q genotype. The secondary aim of this study was to evaluate the E148Q variant and concomitant AA amyloidosis secondary to FMF after adjusting the clinical-demographic characteristics. Further analysis was performed to describe the characteristics of patients who died during the follow-up.

## 2. Materials and Methods

### 2.1. Patients

In this prospective cohort study, patients were recruited from the renal unit at Epigenetic Health Center and the Gulhane School of Medicine in Ankara, Turkey between September 2003 and February 2020. Data of the patients with chronic kidney disease (CKD) had been entered into a detailed registry as described in our previous work [10]. Patients diagnosed with FMF-related AA amyloidosis were included in our registry. Tel-Hashomer criteria were applied in the diagnosis of FMF. All patients had detailed baseline assessment of clinical features, renal functions, genetic testing, histopathological diagnosis of amyloidosis, and treatment received. Patients were evaluated according to the age of FMF diagnosis (<18 years old and ≥18 years old) [11] and E148Q status (homozygous and compound heterozygous). The local ethics committee (GMMA (50687469-1491-70), Ankara, Turkey) approved the study protocol and written informed consent was obtained from each participant. The study was performed according to the principles of the Declaration of Helsinki.

### 2.2. Clinical and Genetic Information

The clinical characteristics of FMF patients including the pattern of episodes, clinical manifestations (skin, musculoskeletal and serosal involvement), constitutional symptoms (including fever, fatigue, headache, malaise, and mood disorders), and medication history were recorded by the physician at the time of registry entry. Body mass index (BMI), systolic (SBP), and diastolic (DBP) blood pressure were measured.

Genetic screening: the reverse-hybridization method (Vienna Lab Diagnostics, Vienna, Austria) was used for molecular diagnosis of FMF. This assay works on the principle that the wild type and mutant type-specific oligonucleotide and target DNA are hybridized. With using streptavidin-alkaline phosphatase, the bound biotinylated sequences are identified as wild-type or mutant-type [12].

Twelve variants in the *MEFV* gene: E148Q, P369S, F479L, M680I (G > C, G > A), I692 del, M694V, M694I, K695R, V726A, A744S, R761H were genotyped. For this purpose, DNA extraction from peripheral blood lymphocytes samples was performed using the QIAamp DNA mini kit (Qiagen, Hilden, Germany). Assay results were evaluated with web-based StripAssay Online Calculator software.

Diagnosis of AA amyloidosis: A kidney biopsy was performed in all patients and the diagnosis of AA amyloidosis was made histologically. The presence of amyloid in tissue sections was confirmed with the positive staining pattern with Congo red dye. Positive and negative controls were used for the diagnosis of AA amyloidosis.

### 2.3. Statistical Analysis

Statistical analysis was performed with Statistical Package of Social Science (SPSS) for Windows, version 15.0 (SPSS Inc., Chicago, IL, USA). The normal distribution was investigated with the Kolmogorov-Smirnov test and histograms. Descriptive statistics were represented as frequencies and percentages for categorical variables and mean ± standard deviation or median (minimum, maximum) for continuous variables as appropriate. A Chi-square test was used to compare demographical and clinical findings between groups. Current age, age at diagnosis of FMF and amyloidosis, and follow-up durations were compared with the Kruskal-Wallis test. A *p*-value < 0.05 was considered statistically significant.

## 3. Results

Our registry consisted of 195 patients with a diagnosis of AA amyloidosis. Of these, 169 patients with complete information (lost to follow-up, *n* = 26) and biopsy-proven FMF-related renal amyloidosis were included. The median age was 36 (19–49) years; the male/female ratio was 1.6 (104/65). The median follow-up duration was 15.0 years (4–17 years). All patients used colchicine and 122 (72.2%) of them were still on colchicine treatment at the last visit.

There were 101 patients diagnosed with FMF < 18 years of age (Group 1) and 68 patients diagnosed ≥ 18 years of age (Group 2). Family history of FMF was documented in 45 patients (44.6%) in group 1 and 11 patients (16.2%) in group 2 (*p* < 0.001). Family history of amyloidosis was reported in 34 patients (33.7%) in group 1, and 7 patients (10.3%) in group 2 (*p* = 0.001) (Table 1). The three most common clinical manifestations were fever (86.1% in group 1, 82.4% in group 2), abdominal pain (71.3% in group 1, 72.1% in group 2), and arthritis (69.3% in group 1, 63.2% in group 2). There was no significant difference between the groups in terms of these clinical manifestations (*p* > 0.05) (Table 1). During the follow-up, 5 patients started dialysis treatment, 9 patients had kidney transplantation. Groups were similar according to BMI, blood pressure, proteinuria, albumin, and glomerular filtration rate (GFR) (*p* > 0.05). The most frequent genotypes were M694V/M694V (45%), M694V/E148Q (14.8%) and E148Q/E148Q (11.2%). Groups were not different according to the frequency of genotypes in the *MEFV* gene (*p* = 0.46) (Table 1).

**Figure 1:**
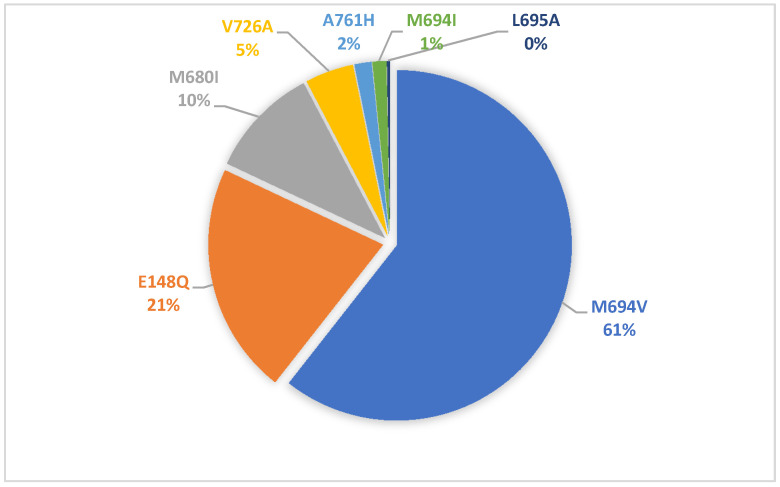
Allele frequencies in MEFV gene in patients with familial Mediterranean fever (FMF)-related renal amyloidosis.

The most common alleles across groups were M694V (60.6%), E148Q (21.4%), and M680I (10.3%) (Figure 1).

Patients with homozygous E148Q mutation were similar in terms of demographic and clinical characteristics to those with compound heterozygous E148Q and homozygous M694V mutation (Table 2). The three most common clinical findings were fever (84.2%), abdominal pain (78,9%), and arthritis (78.9%) in patients homozygous for E148Q, whereas these three, in decreasing order of frequency, were fever (89.3%), abdominal pain (75.0%), and chest pain (60.7%) in patients with compound heterozygous for E148Q (Table 2). Arthritis was less frequent in patients with compound heterozygous for E148Q (46.4%) compared with patients with homozygous E148Q (78.9%) and homozygous M694V mutation (76.3%) (*p* = 0.008). Among the 19 patients with homozygous E148Q mutation, 12 (63.2%) were diagnosed with amyloidosis concurrently with FMF.

Fifteen patients (male/female: 10/5) died during the follow-up (Table 3). Of these 15 patients, 12 died from myocardial infarction and 3 from arrhythmia. Cardiovascular disease (CVD) mortality was almost two times higher in patients with childhood-onset FMF (*n* = 11) than adult-onset (*n* = 4). In those that died, 14 patients were homozygous for M694V and one patient was homozygous for E148Q mutation.

## 4. Discussion

The most serious complication of sustained systemic inflammation in FMF is AA amyloidosis, which can lead to chronic kidney disease. Renal AA amyloidosis with end-stage renal disease is strongly associated with excess mortality in individuals with FMF [10]. The M694V allele and homozygosity were the most common genetic abnormality in our cohort followed by E148Q. During the follow-up period, 15 patients died due to cardiovascular events.

Arthritis is one of the most important clinical features which is associated with more severe disease courses and also more frequently seen in patients with FMF-related AA amyloidosis [5]. It was reported in 47.4% of FMF patients in a Turkish study [13]. In our cohort, arthritis was seen in 66.9% of patients with FMF-related amyloidosis, which is in line with the 66% frequency in a recent study including FMF patients with persistent systemic inflammation [4].

It is well known that the M694V variant is highly associated with amyloidosis [6,7]. The homozygosity of M694V is reported to be the main genetic risk factor for AA-amyloidosis among Non-Ashkenazi Jewish, Turkish, Arab, and Armenian patients in several studies [2,13]. In our cohort, the M694V allele (60.6%) and M694V homozygosity (45.0%) were the most prevalent genotypes. In a retrospective study of 2246 FMF patients, the rate of AA amyloidosis was 8.6% and the M694V variant in the homozygous state was the most common genotype (45.6%) among the AA amyloidosis group [6]. Likewise, M694V homozygosity was observed in 47% of a Turkish FMF cohort with 400 patients with FMF amyloidosis [3]. Our result also supports the M694V variant as a genetic risk factor for the occurrence of renal AA-amyloidosis, as described by others.

The pathogenic role of E148Q, which is one of the most frequent variants of the *MEFV* gene, has been still remained debatable. Ben-Chetrit et al. reported E148Q as a non-disease-causing alteration for the Israel population [14]. E148Q variant frequency in a healthy Turkish population was reported as 12% [15]. In contrary to this finding, some studies demonstrated that patients homozygous for E148Q displayed FMF phenotype [16]. Recent publications from Turkey reported that patients homozygous for the E148Q variant show full clinical features of FMF and E148Q may be a risk for a moderate/severe disease activity [17,18]. Besides, among two different cohorts of AA amyloidosis patients, a total of 9 patients harbored heterozygous E148Q genotype [3,19]. The second most frequent allele was E148Q in 22 patients who presented with proteinuria and eventually, diagnosed with FMF [20]. Of note, 19 out of 169 patients were homozygous for E148Q in our cohort with AA amyloidosis. Contrary to popular belief, our results demonstrate that E148Q/E148Q genotype causes clinical manifestations of FMF, but more importantly it is also associated with AA amyloidosis. Recent data showed that Armenian patients with heterozygous or homozygous E148Q carriers display clinically overt FMF [21]. These findings demonstrate that revisiting the role of E148Q on phenotype and amyloidosis in FMF patients is mandatory to understand whether it is a disease-causing variant or not. We have previously reported the impact of the environment on the disease severity and also the country of residence as a risk factor for the development of amyloidosis [22]. Country of recruitment points to a possible environmental origin of amyloidosis susceptibility. Vigilance is required for clinicians, who work in FMF prevalent regions and follow patients with this genotype, for the possibility of subtle clinical symptoms, ongoing subclinical inflammation, and eventually amyloidosis.

There are limited data on long-term comorbidities and mortality among patients with FMF. Twig et al. reported that during 30 years of follow-up, death rates were 8.73/104 person-years in the FMF group, which was higher than healthy controls [23]. Increased CVD risk in patients with FMF may be related to chronic inflammation [22]. Despite the widespread use of colchicine, amyloidosis remains a significant risk factor for mortality in patients with FMF [6]. The mortality rate in our cohort was 8.9%, most of whom were due to myocardial infarction. Of the 15 dead patients, 14 were homozygous for M694V, which shows the severe disease course in patients with M694V in our cohort. Sustained systemic inflammation and proteinuria are considered among the most important factors for this increased mortality risk. Our previous study also revealed that vascular inflammation and endothelial dysfunction are the main contributors to CVD events in this patient population.

Our study has several strengths and limitations. Well-monitored follow-up data enabled us to evaluate survival. Due to the nature of our registry, we did not recruit FMF patients without amyloidosis. Therefore, we could not define predictors for the development of AA amyloidosis in our cohort. Most of the patients were diagnosed with FMF concurrently with the amyloidosis, hence we were not able to define the time to develop amyloidosis. Another limitation of our study is that the kidney biopsies were recorded without activity and chronicity index to our registry. Mutational screening technique with Strip Assay is not comprehensive and able to screen just known mutational hotspots but, it was the only available method when the registry was established. We were not able to get a myocardial biopsy or postmortem cardiac evaluation to rule out cardiac amyloidosis.

## 5. Conclusions

In conclusion, renal disease is the most important determinant for the prognosis in patients with FMF who develop AA amyloidosis. These patients have an increased risk to develop cardiovascular events and mortality. These patients should be closely monitored for cardiovascular disease, particularly those with modifiable risk factors should be treated aggressively to mitigate the risk of premature mortality. Our results also confirm the strong association between M694V and FMF-associated amyloidosis, which has been reported in many studies. Novel to our study is the higher rate of E148Q homozygosity in patients with amyloidosis. Close clinical monitoring and further evaluation are warranted for patients with the E148Q genotype, particularly for those residing in FMF endemic areas. The possible relationship between E148Q and AA amyloidosis needs to be confirmed in larger prospective cohorts.

## Figures and Tables

**Table 1:** Demographic characteristics and clinical findings, at the time of enrollment, in patients with familial Mediterranean fever (FMF)-related renal amyloidosis, and their comparison according to the age of diagnosis of FMF.

	Age of Diagnosis < 18 (*n* = 101)	Age of Diagnosis ≥ 18 (*n* = 68)	Total (*n* = 169)	*p*
Age Median (min-max) (years)	35 (20–49)	38 (19–45)	36 (19–49)	0.08
Age of diagnosis for FMF Median (min-max) (years)	13.9 (5–17)	20 (18–25)	16 (5–25)	<0.001
Age of diagnosis for amyloidosis Median (min-max) (years)	21 (13–31)	20 (18–25)	20 (13–31)	0.86
Follow-up duration Median (min-max) (years)	13 (4–17)	16 (4–17)	15 (4–17)	0.17
Male *n* (%)	65 (64.4)	39 (57.4)	104 (61.5)	0.35
History of FMF *n* (%)	45 (44.6)	11 (16.2)	56 (33.1)	<0.001
History of Amyloidosis *n* (%)	34 (33.7)	7 (10.3)	41 (24.3)	0.001
Death *n* (%)	11 (10.9)	4 (5.6)	15 (8.9)	0.26
Clinical Findings *n* (%)				
Fever	87 (86.1)	56 (82.4)	143 (84.6)	0.52
Abdominal pain	72 (71.3)	49 (72.1)	121 (71.6)	0.91
Arthritis	70 (69.3)	43 (63.2)	113 (66.9)	0.41
Chest pain	65 (64.4)	36 (52.9)	101 (59.8)	0.13
Arthralgia	45 (44.6)	36 (52.9)	81(47.9)	0.28
Vomiting	30 (29.7)	22 (32.4)	52 (30.8)	0.73
Constipation	21 (20.8)	16 (23.5)	37 (21.9)	0.67
Diarrhea	23 (22.8)	9 (13.2)	32 (18.9)	0.12
Mood disorder	26 (25.7)	14 (20.6)	40 (23.7)	0.43
Myalgia/myositis	27 (26.7)	19 (27.9)	46 (27.2)	0.86
Protracted-febrile-myalgia	17 (16.8)	9 (13.2)	26 (15.4)	0.52
Fatigue	12 (11.9)	10 (14.7)	22 (13.0)	0.59
Headache	10 (9.9)	9 (13.2)	19 (11.2)	0.50
Erysipeloid erythema	7 (6.9)	3 (4.4)	10 (5.9)	0.74 *
Genotypes in *MEFV n* (%)				
M694V/M694V	48 (47.5)	28 (41.2)	76 (45.0)	0.46
M694V/E148Q	13 (12.9)	12 (17.6)	25 (14.8)	
E148Q/E148Q	9 (8.9)	10 (14.7)	19 (11.2)	
E148Q/other variants	1 (1.0)	2 (2.9)	3 (1.8)	
Other	30 (29.7)	16 (23.6)	46 (27.2)	

* Fisher’s exact test.

**Table 2:** Demographic characteristics, clinical findings, kidney biopsy at the time of enrollment, in patients with FMF-related renal amyloidosis, and their comparison according to E148Q and M694V genotypes.

	E148Q/E148Q (*n* = 19)	E148Q/Other Variants (*n* = 28)	M694V/M694V (*n* = 76)	Total (*n* = 123)	*p*
Age Median (min-max) (years)	37 (22–44)	36 (25–49)	36.5 (19–49)	36 (19–49)	0.65
Age of diagnosis for FMF Median (min-max) (years)	18 (8–24)	17.5 (7–22)	16 (5–25)	16 (5–25)	0.79
Age of diagnosis for amyloidosis Median (min-max) (years)	19 (16–26)	19.5 (16–25)	20 (13–31)	20 (13–31)	0.76
Follow up duration Median (min-max) (years)	16 (4–17)	14.5 (4–17)	16 (4–17)	16 (4–17)	0.96
Gender *n* (%)					
Male	9 (47.4)	17 (60.7)	50 (65.8)	76 (61.8)	0.33
Female	10 (52.6)	11 (39.3)	26 (34.2)	47 (38.2)	
History of FMF *n* (%)	7 (36.8)	8 (28.6)	30 (39.5)	45 (36.6)	0.59
History of Amyloidosis *n* (%)	5 (26.3)	7 (25.0)	19 (25.0)	31 (25.2)	0.99
Death	1 (5.3)	-	14 (18.4)	15 (12.2)	0.016
Clinical Findings *n* (%)					
Fever	16 (84.2)	25 (89.3)	65 (85.5)	106 (86.2)	0.86
Abdominal pain	15 (78.9)	21 (75.0)	55 (72.4)	91 (74.0)	0.83
Arthritis	15 (78.9)	13 (46.4)	58 (76.3)	86 (69.9)	0.008
Chest pain	13 (68.4)	17 (60.7)	47 (61.8)	77 (62.6)	0.84
Arthralgia	8 (42.1)	12 (42.9)	39 (51.3)	59 (48.0)	0.63
Vomiting	4 (21.1)	4 (14.3)	26 (34.2)	34 (27.6)	0.10
Constipation	3 (15.8)	5 (17.9)	15 (19.7)	23 (18.7)	0.91
Diarrhea	2 (10.5)	4 (14.3)	17 (22.4)	23 (18.7)	0.39
Mood disorder	3 (15.8)	3 (10.7)	18 (23.7)	24 (19.5)	0.30
Myalgia/myositis	7 (36.8)	2 (7.1)	20 (26.3)	29 (23.6)	0.04
Protracted-febrile-myalgia	4 (21.1)	4 (14.8)	10 (13.2)	18 (14.6)	0.65
Fatigue	4 (21.1)	2 (7.1)	10 (13.2)	16 (13.0)	0.36
Headache	3 (15.8)	3 (10.7)	7 (9.2)	13 (10.6)	0.65
Erysipeloid erythema	-	2 (7.1)	7 (9.2)	9 (7.3)	0.53

**Table 3:** Characteristics of patients who died during the follow-up period.

Number	Sex	Age of FMF Diagnosis (Years)	Age of Amyloidosis Diagnosis (Years)	Age of Death (Years)	Genotype	Disease Duration (Years)	Cause of Death
1	Female	13	21	36	M694V/M694V	23	Arrhythmia
2	Male	11	23	36	M694V/M694V	25	MI *
3	Male	9	18	36	M694V/M694V	27	Arrhythmia
4	Male	18	18	37	M694V/M694V	19	MI
5	Male	11	21	39	M694V/M694V	28	MI
6	Female	13	22	39	M694V/M694V	26	MI
7	Female	14	18	40	E148Q/E148Q	26	MI
8	Male	7	21	41	M694V/M694V	34	MI
9	Male	11	22	43	M694V/M694V	32	MI
10	Male	21	22	44	M694V/M694V	23	MI
11	Female	18	23	44	M694V/M694V	26	MI
12	Male	14	19	44	M694V/M694V	30	Arrhythmia
13	Male	17	17	45	M694V/M694V	28	MI
14	Male	20	20	47	M694V/M694V	27	MI
15	Female	15	19	49	M694V/M694V	35	MI

* MI; myocardial infarction.

## Data Availability

The data presented in this study are available on request from the corresponding author.
